# *USP38, FREM3, SDC1, DDC,* and *LOC727982* Gene Polymorphisms and Differential Susceptibility to Severe Malaria in Tanzania

**DOI:** 10.1093/infdis/jiv192

**Published:** 2015-03-24

**Authors:** Alphaxard Manjurano, Nuno Sepúlveda, Behzad Nadjm, George Mtove, Hannah Wangai, Caroline Maxwell, Raimos Olomi, Hugh Reyburn, Christopher J. Drakeley, Eleanor M. Riley, Taane G. Clark

**Affiliations:** 1 Joint Malaria Programme,Kilimanjaro Christian Medical College, Moshi; 2 National Institute for Medical Research, Dar es Salaam, Tanzania; 3 Departments ofImmunology and Infection; 4 Clinical Research; 5 Pathogen Molecular Biology; 6 Infectious Disease Epidemiology, London School of Hygiene and Tropical Medicine, United Kingdom; 7 Centre of Statistics and Applications, University of Lisbon, Portugal

**Keywords:** genetic association study, host susceptibility, *Plasmodium falciparum*, severe malaria, Tanzania

## Abstract

Populations exposed to *Plasmodium falciparum* infection develop genetic mechanisms of protection against severe malarial disease. Despite decades of genetic epidemiological research, the sickle cell trait (HbAS) sickle cell polymorphism, ABO blood group, and other hemoglobinopathies remain the few major determinants in severe malaria to be replicated across different African populations and study designs. Within a case-control study in a region of high transmission in Tanzania (n = 983), we investigated the role of 40 new loci identified in recent genome-wide studies. In 32 loci passing quality control procedures, we found polymorphisms in *USP38, FREM3, SDC1, DDC,* and *LOC727982* genes to be putatively associated with differential susceptibility to severe malaria. Established candidates explained 7.4% of variation in severe malaria risk (HbAS polymorphism, 6.3%; α-thalassemia, 0.3%; ABO group, 0.3%; and glucose-6-phosphate dehydrogenase deficiency, 0.5%) and the new polymorphisms, another 4.3%. The regions encompassing the loci identified are promising targets for the design of future treatment and control interventions.

Malaria is one of the most prevalent infectious diseases in the world, with ≥225 million cases and 781 000 deaths per year, most involving *Plasmodium falciparum* infections in children in sub-Saharan Africa [1]. The etiology of malaria is complex, with many genetic and environmental determinants influencing the natural variation in response to infection, progression and severity. Disease phenotypes are influenced by host age, state of immunity, and genetic background, as well as parasite genetic make-up [2].

Heritability studies have estimated that approximately 25% of the risk for severe malaria progression is determined through human genetic factors [2]. The disease has also exerted significant selection pressure on the human genome, as evidenced by the congruence of malaria parasite prevalence with sickle cell trait (HbAS) and other hemoglobinopathies, such as thalassemias and glucose-6-phosphate dehydrogenase (G6PD) deficiency. Despite >20 years of candidate gene studies in severe malaria and its clinical subtypes (eg, cerebral malaria), the HbAS polymorphism remains one of the few determinants to be replicated across different African populations and study designs [3].

In the past decade, the development of high-throughput molecular technologies, the sequencing of the human genome, and progress in understanding human genetic diversity has allowed candidate gene studies to be augmented by unbiased genome-wide discovery approaches. Genome-wide association studies (GWASs) for severe malaria have not only confirmed the effects of HbAS and such candidates as HbAC and the ABO blood group, but they have also identified other loci putatively associated with disease risk [3–6]. These novel loci may affect malaria susceptibility directly or indirectly (through single-nucleotide polymorphism [SNP] correlations or linkage disequilibrium [LD]) by modulation of the immune response and/or interfering with the parasite life cycle inside the host [3–6]. For example, the *DDC* gene and neighboring loci (eg, *IZKF1*) were identified in a GWAS in a Gambian population with a predominantly cerebral malaria case mix [3]. Mutations in *DDC* are the cause of aromatic L-amino acid decarboxylase deficiency, an inborn error in neurotransmitter metabolism that leads to combined serotonin and catecholamine deficiency.

Polymorphisms in the *ATP2B4* locus, encoding a major calcium transporting pump, seems to be protective against severe childhood malaria in Ghana [4] and in a GWAS analysis across Gambian, Kenyan, and Malawian populations [5]. *ATP2B4* is upstream of *LAX1*, a transmembrane protein expressed in peripheral blood lymphocytes and implicated in T and B-cell responsiveness [5]. Additional work in Ghana has shown protective effects of *ATP2B4* on malaria in pregnancy and related maternal anemia, suggesting that *ATP2B4* variant-associated protection is not limited to severe childhood malaria [7]. The 3 population GWAS analysis also revealed a putative region centered on *SMARCA5* in chromosome 4 [5], 250 kb upstream of the *GYPE/A/B* gene cluster encoding the MNS blood group system, known putative receptors for the *P. falciparum* parasite. This genomic region, including *INPP4B, USP38, GAB1, GUSBP5, FREM3, and GYPB* loci has been implicated as being under ancient selective pressure, with particularly strong signals for the *FREM3 locus,* which is in almost perfect LD with *GYPB and GYPA* [8].

Other candidates are being identified through the involvement of common genetic pathways in susceptibility to, or protection from, a number of different infectious diseases. For example, the major histocompatibility complex genes play a central role in the immune response to pathogens and self antigens, and the *HECW* locus has been identified in *Caenorhabditis elegans* as affecting pathogen avoidance behavior [9]. In contrast, other candidates found in GWAS association or selection analysis have no apparent previous link to infectious disease (eg*, OXNAD1, LOC727982, SDC1, ZSWIM2,* and *LPHN2)* [6].

In the current study, we investigated the role of 114 polymorphisms in 40 new candidate loci, including *DDC* and *FREM3*, identified from previous GWASs [3–6]. As part of a MalariaGEN consortial project described elsewhere [6], genotyping assays were developed for 114 SNPs at these loci and typed in a case-control study of severe malaria (n = 983) conducted in an intense malaria transmission setting in Tanga, Tanzania [10, 11]. Previous work in the same individuals using 68 SNPs across well-documented malaria candidate genes (eg, HbAS, G6PD, major histocompatibility complex class III loci) revealed only the HbAS polymorphism to be associated strongly with protection from all forms of severe malaria (*P* < 10^−8^) [10].

There were some weaker X chromosome-specific associations, with protection from severe malaria phenotypes due to the G6PD A- alleles (202A/376G) in females. More extensive genotyping of the *G6PD* locus revealed that the observed female heterozygous advantage is due to balancing selection [11]. Similarly, 2 SNPs in the gene encoding the CD40 ligand (*CD40L,* X chromosome) were associated with severe malarial anemia (rs3092945) and respiratory distress (rs1126535) in females, but not through heterozygous advantage, and not in the cohort overall. The importance of the *CD40L* locus, as well as *ABO* (O group, rs8176719)*, ATP2B4* (rs10900585), and *G6PD* (rs1050828), has been confirmed in a meta-analysis across African populations (*P* < 10^−4^) [12]. In the same multicenter study, the other 22 candidates considered previously in the Tanzanian study did not reach statistical significance overall.

Using the new candidate polymorphisms, we first set out to establish whether any were significantly associated with severe malaria phenotypes in Tanzania. We then quantified the contribution of these loci to protection from severe malaria over and above that conferred by the established candidate polymorphisms, such as HbAS. We found 9 novel SNPs associated with protection from severe malaria, some affecting different clinical subtypes, such as cerebral malaria and severe malarial anemia. These polymorphisms explain an additional 4.3% of the variation in risk of severe malaria (and 19.2% of the explainable variation) over and above the combined 7.4% accounted for by the HbAS polymorphism (6.3%), α-thalassemia (0.3%), ABO blood group (0.3%), and G6PD (0.5%) genes. The loci identified provide a rich area for further investigation and potential new avenues for developing malaria treatment and control measures.

## METHODS

### Study Participants and Phenotypes

The study was conducted in Teule district hospital and surrounding villages in Tanga region, Tanzania. Patients with severe malaria (n = 506), aged 6 months to 10 years, were recruited between June 2006 and May 2007, if they fulfilled any of the following eligibility criteria: history of ≥2 convulsions in the last 24 hours, prostration (unable to sit unsupported if <9 months of age or unable to drink at any age), reduced consciousness (Blantyre coma scale score, <5), respiratory distress, jaundice, severe malarial anemia (hemoglobin, <5 g/dL [measured with HemoCue system]), acidosis (blood lactate, >5 mmol/L), or hypoglycemia (blood glucose, <2.5 mmol/L). Patients were defined as having had cerebral malaria if their Blantyre coma scale score was ≤3. Parasite infection was assessed with rapid diagnostic test (ParaScreen Pan/Pf test for horseradish peroxidase 2) and double-read Geimsa-stained thick blood films. Controls (n = 477) without any recorded history of severe malaria were matched for ward of residence, ethnicity, and age, using household lists during a 4-week period in August 2008. Study participants resided in 33 geographic wards (Mtindiro, 9.6%; Kwafungo, 8.5%; Mkata, 6.3%; Kwedizinga, 6.0%; others, each <5.0%) in Tanga region and were predominantly from 7 ethnic groups (Table 1).

**Table 1.** Baseline and Clinical Characteristics

Characteristic	No. (%)^a^		Difference *P* Value
	Controls (n = 477)	Cases (n = 506)	
Age, median (range), mo	2.9 (0.9–10.9)	1.7 (0.2–10.0)	<.0001
Female sex	255 (53.5)	236 (46.6)	.04
Ethnicity			.0005
Mzigua	158 (33.1)	149 (29.4)	
Wasambaa	130 (27.3)	130 (25.7)	
Wabondei	78 (16.4)	79 (15.6)	
Chagga	50 (10.5)	47 (9.3)	
Mmbena	23 (4.8)	25 (4.9)	
Mngoni	18 (3.8)	19 (3.8)	
Pare	15 (3.1)	18 (3.6)	
Other	5 (1.0)	39 (7.7)	
Blood group			.22
O	236 (50.1)	216 (44.4)	
A	115 (24.4)	145 (29.8)	
B	100 (21.2)	10 (22.0)	
AB	20 (4.2)	18 (3.7)	
Sickle HbAS (rs334)^b^			<.0001
AA	385 (83.5)	473 (97.9)	
AS	76 (16.5)	5 (1.0)	
SS	0 (0.0)	5 (1.0)	
α-thalassemia			.15
αα/αα	224 (47.2)	236 (51.9)	
αα/α-	199 (41.9)	184 (40.4)	
α-/α-	52 (10.9)	35 (7.7)	
G6PD G202A (female)			.01
GG	154 (62.6)	159 (71.0)	
AG	83 (33.7)	52 (23.2)	
AA	9 (3.7)	13 (5.8)	
A	51 (20.5)	39 (17.4)	
G6PD202A (male)	41 (19.3)	37 (14.7)	.23
G6PD A376G (female)			.05
AA	83 (33.5)	106 (47.1)	
AG	133 (53.6)	85 (37.8)	
GG	32 (12.9)	34 (15.1)	
G	99 (39.7)	77 (34.0)	
G6PD376G (male)	79 (37.1)	90 (36.7)	.98
Any severe malaria	…	506 (100)	…
Any severe malarial anemia^b^	…	246 (48.6)	…
Any cerebral malaria	…	99 (19.6)	…
Both severe malarial anemia and cerebral malaria	…	41 (8.1)	…
Any respiratory distress	…	146 (28.9)	…
Acidosis^c^	…	291 (57.5)	…

Abbreviation: HbAS, sickle cell trait.

^a^ Unless otherwise specified, data represent No. (%) of patients or controls.

^b^ Defined as hemoglobin <5 gdL.

^c^ Defined as blood lactate >5 mmol/L.

### Sample Genotyping

DNA was extracted from blood samples as described elsewhere [10]. The MalariaGEN resource center identified candidate SNPs by analyzing multiple GWASs of severe malaria [3, 4, 6] and used the Sequenom iPlex platform [13] to genotype these loci, as part of a multicenter consortial study described elsewhere [6]. The genotyping assays included 114 SNP positions across 40 loci (Supplementary Table 1). Genotyping data from the previously published 68 SNPs, including HbAS (rs334), HbAC (rs33930165), HbE (rs33950507), and ABO blood group (rs8176719 and rs8176746) were used for comparisons [10, 11]. The α^3.7^-thalassemia deletion was typed separately by polymerase chain reaction [10].

### Statistical Analysis

Deviations in genotypic frequencies from Hardy–Weinberg equilibrium were assessed using χ^2^ analysis. SNPs were excluded from analysis if ≥10% of genotype calls were missing, if there was significant deviation from Hardy–Weinberg equilibrium in autosomal SNPs (*P* < .0001) in controls, or if the overall minor allele frequency was <0.01. Using these criteria, 25 SNPs (8 loci) were removed, leaving 89 for association analysis (see Supplementary Table 1). Case-control association analysis using SNP alleles/genotypes was undertaken using logistic regression and including age, sex, and ethnic group as covariates. We modeled the SNP of interest assuming several related genotypic mechanisms (additive, dominant, recessive, heterozygous advantage, and general models) and reported the minimum *P* value from these correlated tests. Epistatic effects between polymorphisms were considered by inclusion of statistical interactions in these models.

Haplotypes were inferred from genotypes using an expectation-maximization algorithm [14]. Haplotype association testing was performed using the logistic regression models described above [14], and LD was estimated using pairwise *D*′ (and *r^2^*) metrics [15]. To minimize the occurrence of false-positives due to multiple statistical testing, and using a permutation approach that accounted for correlation between markers and tests, we estimated associations to be statistically significant at *P* < .008. In previous work [10], only G6PD A^−^ (376/202) and HbAS polymorphisms were statistically significant at this threshold. The proportion of phenotypic variation explained by individual SNPs was estimated using the modEvA package (version 0.9) on R-Forge. Tajima's *D* metric was used to quantify evidence of balancing selection [16]. All analyses were performed using the R statistical package (http://www.r-project.org).

## RESULTS

The severe malaria case mix (n = 506) included cases of acidosis (57.5%), severe malarial anemia (48.6%), respiratory distress (28.9%), and cerebral malaria (19.6%) (Table 1 and Supplementary Figure 1). Compared with controls (n = 477), case patients with malaria were younger and more likely to be male, with fewer from the 7 main ethnic groups (*P* < .05). As expected, case patients were less likely to be of blood group O (odds ratio [OR] for O vs A, 0.726; 95% confidence interval [CI], .534–.986; *P* = .04), or to have α-/α- thalassemia (α-/α- vs αα/αα or αα/α-, 0.639; .401–1.018; *P* = .06) or sickle cell protective AS (AS vs other, 0.053; .021–.132; *P* < 10^−17^) genotypes. Allele frequencies for both G6PD202A (case patients vs controls, 16.3% vs 20.0%) and G6PD376G (37.4% vs 38.5%) were slightly lower in malaria case patients than in controls (*P* < .02), and any protective effects were seen predominantly in female and not male patients (Table 1). In particular, protection was observed in female heterozygotes with G6PD376 (OR for female AG vs AA/GG, 0.436 [95% CI, .257–.621; *P* = .00007]; male A vs G, 1.012 [.672–1.522; *P* = .956]) or G6PD202 (female AG vs AA/GG, 0.518 [.325–.827; *P* = .005]; male A vs G, 0.774 [.461–1.300; *P* = .333]).

**Figure 1.**
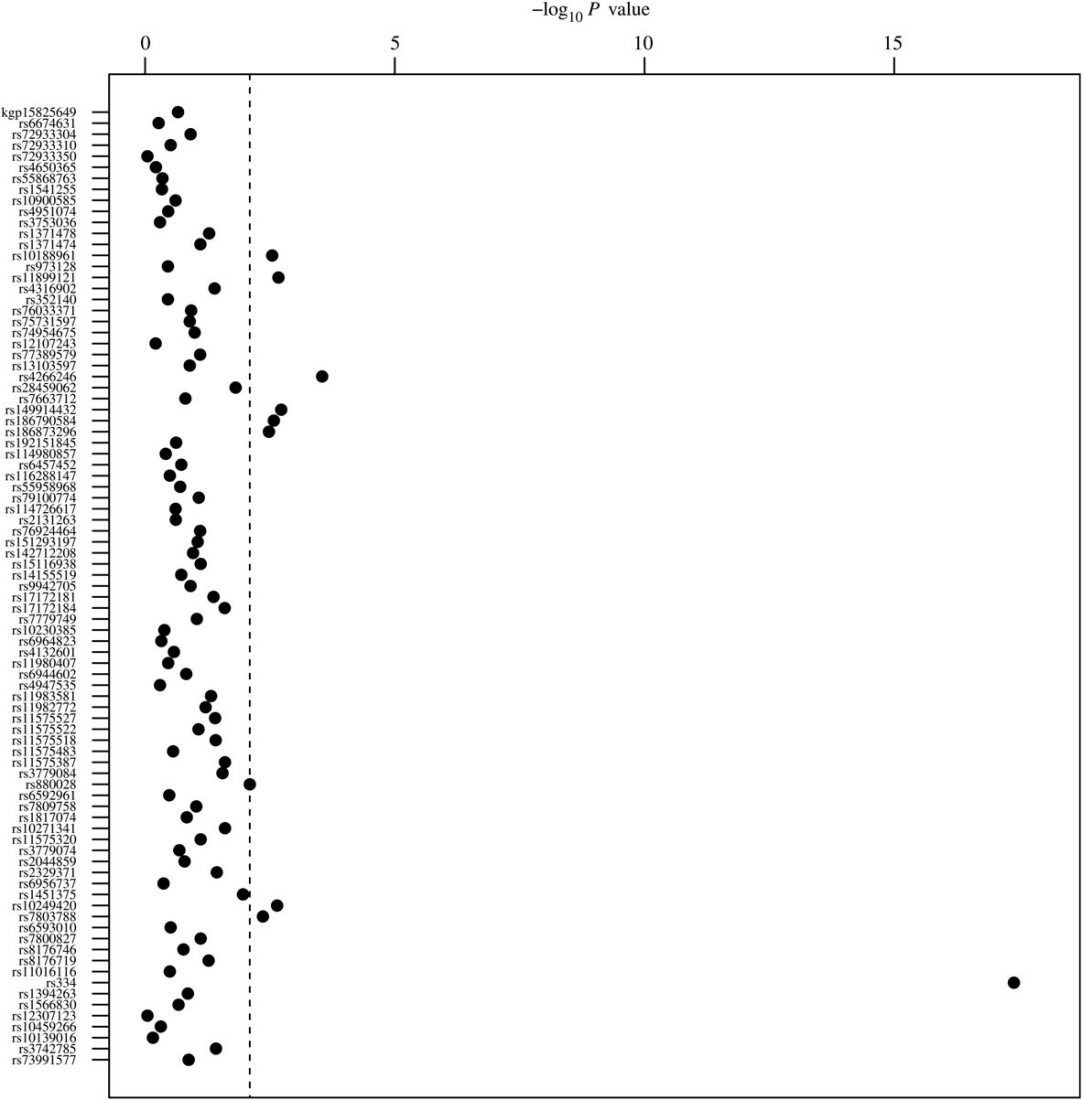
Association *P* values for severe malaria. Vertical dashed line represents *P* value of .008.

Association analysis using the 89 high-quality polymorphisms revealed 10 SNPs (6 loci) associated with severe malaria (Figure 1 and Table 2). These included HbAS (*HBB*; *P* < 10^−17^); rs4266246 (*USP38*; *P* = .0003); rs149914432, rs186790584, and rs186873296 (*FREM3*; all *P* < .0033); rs11899121 (*SDC1*; *P* = .0021); rs10188961 (*LOC727982*; *P* = .0028); and rs10249420, rs7803788, and rs880028 (*DDC*; *P* < .0080). No associations were observed at the *ATP2B4* locus (*P* > .247). The heterozygous advantage effect of the HbAS-AS genotype was statistically significant across all clinical phenotypes (OR, 0.021–0.056; *P* < 10^−6^) whereas the rs4266246 (*USP38*) association was driven by approximately 50% increased susceptibility to acidosis (OR for additive T allele, 1.549; *P* = .0015) and severe malarial anemia (OR for additive T allele, 1.443; *P* = .0048).

**Table 2.** Severe Malaria Associations

Phenotype and SNP	Gene Name	Allele		MAF		Genotype Comparison	OR (95% CI)^a^	*P* Value
		Major	Minor	Control	Case			
Severe malaria								
rs334	*HBB*	A	S	0.082	0.016	AS vs other	0.056 (.022–.142)	4.0 × 10^−18^
rs4266246	*USP38*	C	T	0.225	0.313	CT/TT vs CC	1.665 (1.263–2.195)	.0003
rs149914432	*FREM3*	A	C	0.060	0.031	AC vs other	0.447 (.265–.752)	.0019
rs11899121	*SDC1*	C	G	0.479	0.403	CG/GG vs CC	0.629 (.467–.847)	.0021
rs10249420	*DDC*	C	G	0.152	0.108	CG vs other	0.605 (.438–.837)	.0023
rs186790584	*FREM3*	A	T	0.056	0.030	AT vs other	0.452 (.266–.768)	.0026
rs10188961	*LOC727982*	A	G	0.354	0.415	Additive G	1.359 (1.110–1.665)	.0028
rs186873296	*FREM3*	A	G	0.055	0.030	AG/GG vs AA	0.471 (.280–.793)	.0033
rs7803788	*DDC*	C	T	0.152	0.112	CT vs other	0.628 (.456–.866)	.0044
rs880028	*DDC*	A	G	0.167	0.181	AG vs other	1.497 (1.105–2.026)	.0080
Severe malarial anemia								
rs334	*HBB*	A	S	0.082	0.021	AS vs other	0.047 (.011–.199)	5.2 × 10^−11^
rs4266246	*USP38*	C	T	0.225	0.332	Additive T	1.549 (1.182–2.031)	.0015
rs6457452	*HSPA1B*	C	T	0.237	0.177	Additive T	0.661 (.486–.900)	.0073
Cerebral malaria								
rs334	*HBB*	A	T	0.082	0.000	AS vs other	0.000 (NA)	1.5 × 10^−7^
rs10249420	*DDC*	C	G	0.152	0.077	CG vs other	0.409 (.213–.786)	.0040
Respiratory distress;								
rs334	*HBB*	A	S	0.082	0.007	AS vs other	0.086 (.020–.366)	2.5 × 10^−6^
rs11899121	*SDC1*	C	G	0.479	0.366	CG/GG vs CC	0.505 (.325–.784)	.0025
Acidosis								
rs334	*HBB*	A	T	0.082	0.012	AS vs other	0.021 (.003–.151)	9.8 × 10^−14^
rs10188961	*LOC727982*	A	G	0.354	0.429	Additive G	1.488 (1.169–1.894)	.0011
rs4266246	*USP38*	C	T	0.225	0.314	Additive T	1.443 (1.117–1.862)	.0048
rs149914432	*FREM3*	A	C	0.060	0.030	AC vs other	0.411 (.211–.798)	.0057

Abbreviations: CI, confidence interval; MAF, minor allele frequency; NA, cannot be estimated; OR, odds ratio; SNP, single-nucleotide polymorphism.

^a^ All ORs were adjusted for age, ethnicity, and sex.

For the other severe malaria associations there were only significant signals for single clinical phenotypes. The rs11899121 (*SDC1*) SNP was associated with a approximately 49% reduction in risk of respiratory distress (OR for CG/GG vs CC genotypes, 0.505; *P* = .0025). Similarly, rs10188961 (*LOC727982*) was associated with increased susceptibility to acidosis (OR for additive G allele, 1.488; *P* = .0011). The most significant association—between severe malaria and *FREM3* (rs149914432)—arose through a approximately 59% reduced risk of acidosis (OR for AC vs other genotypes, 0.411; *P* = .0057). The other putative *FREM3* severe malaria associations did not reach the statistical significance threshold for the acidosis phenotype (rs186790584, *P* = .009; rs186873296, *P* = .010). The *DDC* association arose through a heterozygous protective effect against cerebral malaria (OR, 0.409; *P* = .0040). Whereas the rs6457452 SNP (*HSPA1B*) was associated with 34% reduced risk of severe malarial anemia (OR for additive T allele, 0.661; *P* = .0073), there was weaker evidence for an effect on severe malaria overall (*P* = .010).

**Figure 2.**
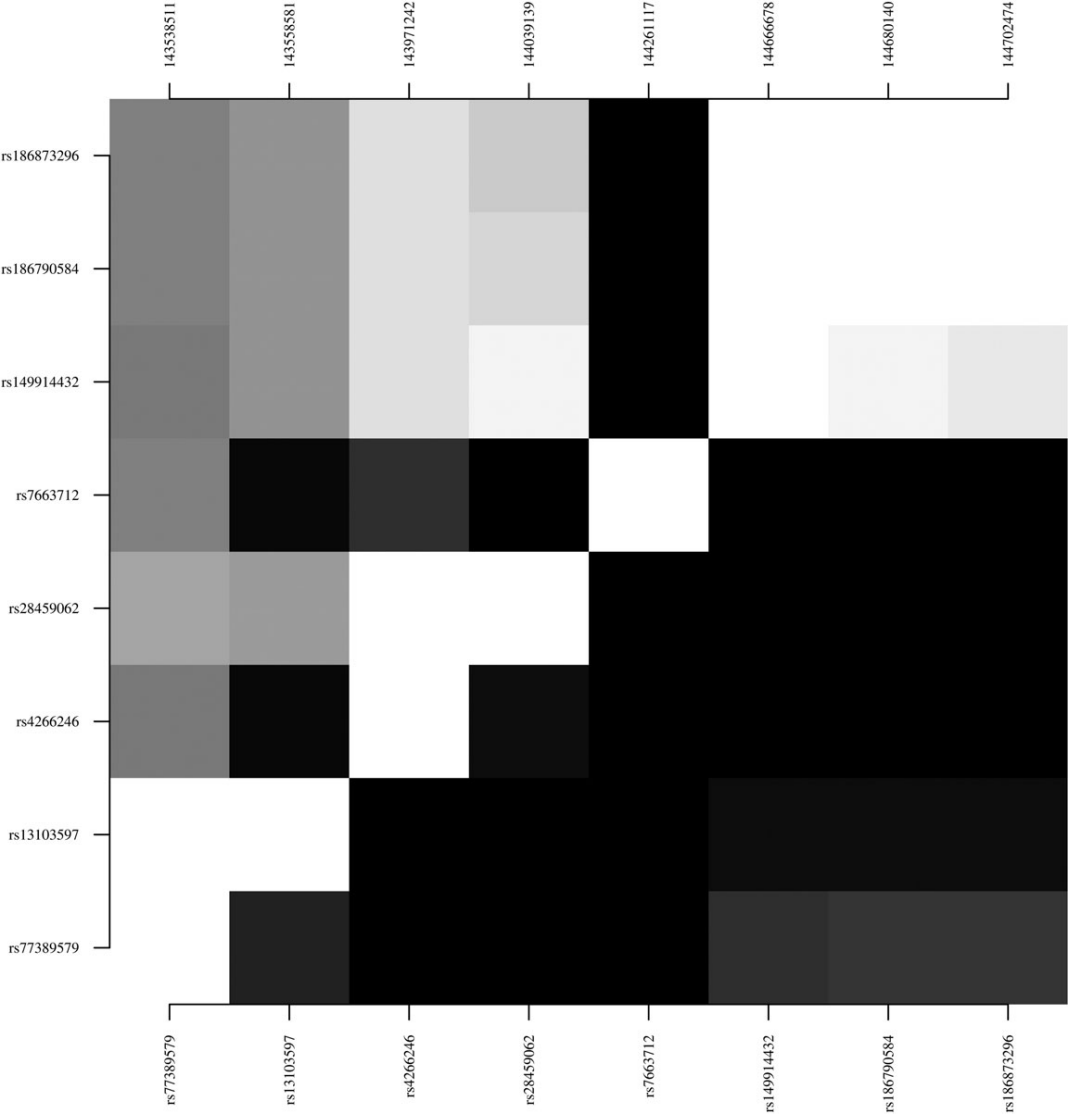

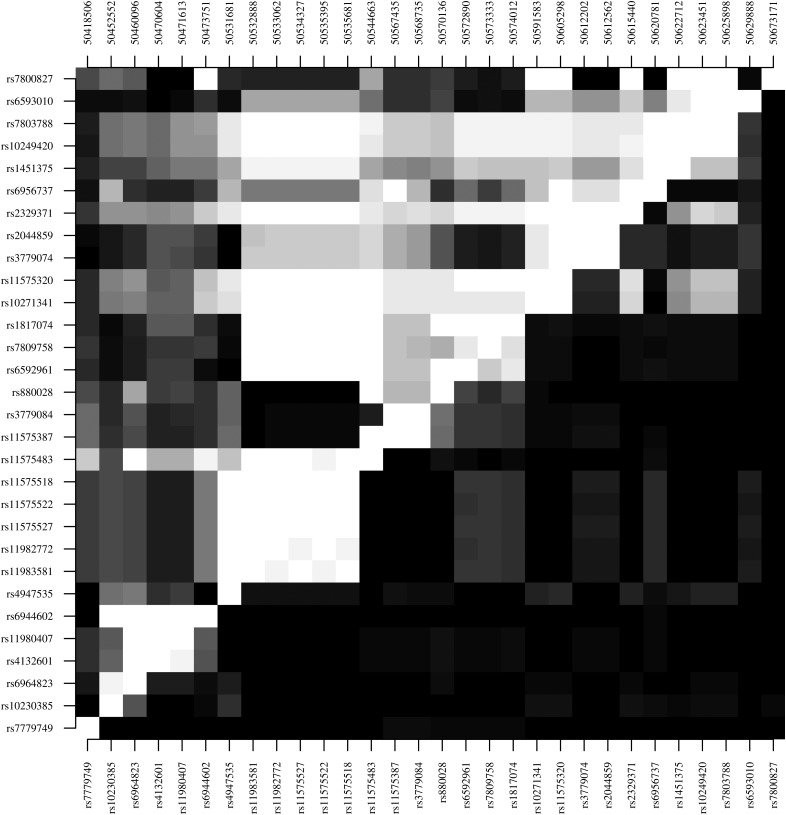
Linkage disequilibrium, Top left, *D′*; bottom right, *r*^2^. Black represents 0; white, 1. *A*, Single-nucleotide polymorphisms (SNPs) in *FREM3* and flanking candidate genes on chromosome 4: rs77389579 and rs13103597 (*INPP4B*), rs4266246 and rs28459062 (*USP38*), rs7663712 (*GAB1*), and rs149914432, rs186790584, and rs186873296 (*FREM3*). *B*, SNPs in the *DDC* and *IZKF1* genes on chromosome 7: rs7779749, rs10230385, rs6964823, rs11552046, rs4132601, rs11980407, and rs6944602 (*IZKF1*) and rs4947535–rs7800827 (*DDC*).

For the 2 loci with multiple association hits (*FREM3* [3 association hits] and *DDC* [3 association hits]), we assessed the level of LD within and flanking the gene to inform a haplotype-based analysis. For the chromosome 4 region containing *FREM3* and *USP38* (length, 1.16 megabase pairs), there was high LD within the genes (minimum *D*′*,* 0.998 for *FREM3*, 0.998 for *USP38,* and 0.999 for *INPP4B*) and some limited evidence that this linkage extended to SNPs within different genes (maximum *D*′, 0.893 between *FREM3* and *USP38*) (Figure 2*A*). Similarly, for the genomic region on chromosome 7 containing *DDC* and *IZKF1* (length, 255 kilobase pairs), there were several high-LD blocks , including 2 covering the association hits (block 1: rs880028, rs6592961, rs7809758, and rs1817074 [median *D*′, 0.973]; block 2: rs10249420 and rs7803788 [*D*′, 1.000]) (Figure 2*B*).

A haplotype analysis using the *FREM3* SNPs (rs149914432, rs186790584, and rs186873296) revealed a 55% reduced risk of severe malaria between the 2 major haplotypes (OR for CTG vs AAA, 0.448; *P* = .003) (Table 3). Further analysis accounting for the genotypic combinations of haplotypes revealed that AAA /CTG heterozygotes were protected compared with AAA homozygotes (wild-type) (OR, 0.450; *P* = .0032) (Table 4). There was some evidence of the combined haplotype effect on acidosis (OR, 0.423; 95% CI, .217–.825; *P* = .012). These results are consistent with the heterozygous advantage effects from the single SNP analyses.

**Table 3.** Haplotype Analysis and Severe Malaria Phenotypes

Phenotype and Gene	Haplotype	Frequency			*P* Value
		Control	Case	OR (95% CI)^a^	
Severe malaria					
* FREM3*^b^	AAA	0.941	0.969	1.000	…
	CTG	0.053	0.024	0.448 (.266–.756)	.006
* DDC* block 1^c^	AGAT	0.642	0.613	1.000	…
	AAGC	0.175	0.191	1.100 (.855–1.417)	.458
	AGGC	0.013	0.015	1.580 (.682–3.661)	.286
	GAAC	0.027	0.019	0.664 (.342–1.291)	.228
	GAGC	0.136	0.160	1.277 (.962–1.696)	.091
* DDC* block 2^d^	CC	0.847	0.887	1.000	…
	GT	0.152	0.113	0.724 (.545–.963)	.027
Acidosis					
* FREM3*^b^	AAA	0.941	0.971	1.000	…
	CTG	0.053	0.026	0.479 (.266–.860)	.015
Cerebral malaria					
* DDC* block 1^c^	AGAT	0.642	0.606	1.000	…
	AAGC	0.175	0.200	1.215 (.822–1.795)	.421
	AGGC	0.013	0.016	1.242 (.341–4.516)	.398
	GAAC	0.027	0.015	0.634 (.186–2.165)	.382
	GAGC	0.136	0.163	1.291 (.838–1.988)	.298
* DDC* block 2^d^	CC	0.847	0.914	1.000	…
	GT	0.152	0.086	0.531 (.300–.940	.030

Abbreviations: CI, confidence interval; OR, odds ratio.

^a^ All ORs were adjusted for age, ethnicity, and sex.

^b^ *FREM3*: rs149914432, rs186790584, and rs186873296.

^c^ *DDC* block 1: rs880028, rs6592961, rs7809758, and rs1817074.

^d^ *DDC* block 2: rs10249420 and rs7803788.

**Table 4.** Severe Malaria and Combinations of Haplotypes

Gene	Haplotypes	Controls, %	Case Patients, %	OR (95% CI)^a^	*P* Value
*FREM3*^b^	1, 1	88.4	94.2	1.000	…
	1, 2	10.7	5.2	0.450 (.265–.765)	.003^c^
	1, 3	0.6	0.2	0.417 (.035–4.977)	.490
	2, 2	0.2	0.4	0.856 (.074–9.875)	.901
*DDC*					
Block 1^d^	1, 1	41.9	37.9	1.000	
	1, 2	21.6	22.7	1.074 (.746–1.547)	.700
	1, 3	2.2	1.3	0.836 (.275–2.545)	.753
	1, 4	3.5	2.3	0.668 (.286–1.557)	.350
	1, 5	17.7	21.1	1.348 (.916–1.983)	.129
	2, 2	4.4	4.0	0.945 (.456–1.958)	.878
	2, 5	2.6	5.9	2.794 (1.296–6.024)	.009^c^
	5, 5	2.8	1.5	0.531 (.191–1.475)	.225
	Other	3.3	3.4	1.122 (.515–2.445)	.772
Block 2^e^	1, 1	71.2	78.9		
	1, 2	27.1	19.5	0.643 (0.467–0.885)	.007^c^
	2, 2	1.7	1.6	1.097 (.371–3.242)	.867

Abbreviations: CI, confidence interval; OR, odds ratio.

^a^ All ORs were adjusted for age, ethnicity, and sex.

^b^ *FREM3*: rs149914432, rs186790584, and rs186873296 (haplotype 1 represents AAA; 2, CTG; 3, CAA).

^c^ Significant associations (*P* < .01).

^d^ *DDC* block 1: rs880028, rs6592961, rs7809758, and rs1817074 (haplotype 1 represents AGAT; 2, AAGC; 3, AGGC; 4, GAAC; 5, GAGC).

^e^ *DDC* block 2: rs10249420 and rs7803788 (haplotype 1 represents CC; 2, GT).

Severe malaria haplotype analysis of *DDC* block 1, which includes the rs880028 polymorphism, revealed no significant associations (*P* > .091) (Table 3) although the heterozygous disadvantage of this polymorphism was partially recaptured by considering haplotype combinations (OR for GAGC/AAGC vs AGAT/ AGAT, 2.794; *P* = .0088) (Table 4). However, for patients with cerebral malaria, in whom the frequency of GAGC/AAGC was low (3 in 99), no haplotypic association was seen (OR, 1.148; 95% CI, .296–4.447; *P* = .841). The analysis of severe malaria and DDC block 2 also captured the heterozygous advantage effects of the rs10249420 and rs7803788 polymorphisms when haplotype combinations were considered (OR for GT/CC vs CC/CC, 0.643; *P* = .0068) (Table 4). There was some evidence of this effect only in patients with cerebral malaria (OR for GT/CC vs CC/CC, 0.476; 95% CI, .256−.883; *P* = .0185).

The Tajima's *D* metric was used to determine whether the heterozygous association effects in the *FREM3* and *DDC* loci were due to balancing selection. This seems to be the case for *DDC* (Tajima's *D,* 3.035 for controls vs 2.787 for case patients) but not for *FREM3* (−0.965 vs −1.245), for which increasing positive values are indicative of balancing selection. The magnitude of the *DDC* effect is at the extreme positive tail of an observed negatively centered Tajima's *D* distribution for African populations [17], where predominantly negative values demonstrate either slow growth from a small population size or a bottleneck that predates the migration of the human species out of Africa [17]. Such balancing mechanisms exist at other malaria candidate loci, including the HbAS sickle trait [8] and G6PD [11]. There was no evidence of epistatic effects between HbAS, G6PD, α-thalassemia, and the new candidate loci (*USP38, DDC*, *FREM3*, *SDC1,* and *LOC727982)* for severe malaria risk (*P* > .129).

**Figure 3.**
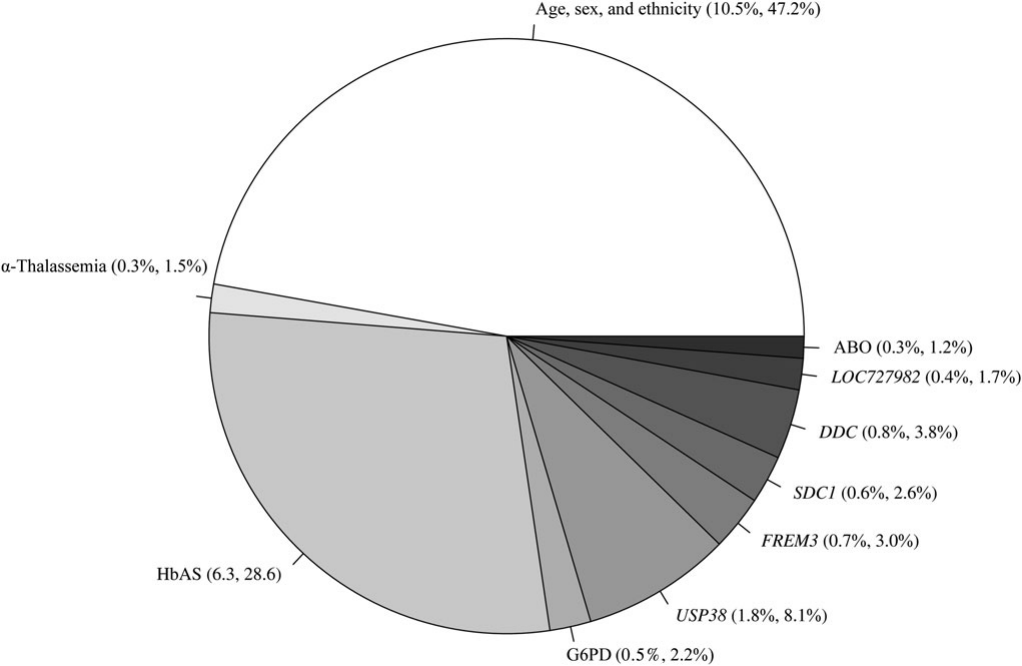
Breakdown of explainable variation in the severe malaria association model (nominal percentage, relative percentage). Abbreviations: G6PD, glucose-6-phosphate dehydrogenase; HbAS, sickle cell trait.

Finally, we assessed the proportion of variation in the severe malaria phenotype that is explained by the new candidate loci (Figure 3). Of the 22.2% of variation explained overall, age, sex, and ethnicity accounted for 47.2%, and the established gene candidates accounted for another 33.6% (HbAS, 28.6%; G6PD, 2.2%; α-thalassemia, 1.5%, ABO blood group, 1.2%). The new candidates accounted for the remaining 19.2% of the explainable variation, with *USP38* (8.1%), *DDC* (3.8%), *FREM3* (3.0%), *SDC1* (2.6%), and *LOC727982* (1.7%) all accounting for more variation than ABO blood group and α-thalassemia but in aggregate less than the sickle HbAS polymorphism. Overall, the newly identified polymorphisms explain an additional nominal 4.3% of the variation in risk of severe malaria over and above the combined 7.4% for HbAS (6.3%), ABO blood group (0.2%), α-thalassemia (0.3%), and G6PD (0.5%).

## DISCUSSION

As noted for historical candidate genes studies, GWAS findings are not always replicated across studies or populations, leading to reporting of false-positive associations and suspicion of the validity of novel associations [18]. In studies of severe malaria in African populations, reproducibility is compromised in part by allelic heterogeneity across ethnic groups [19], differential patterns of LD [18], and the heterogeneity of the phenotype [3]. Associations are further complicated by diversity in *Plasmodium* parasite and *Anopheles* mosquito factors, both genetic and environmental, and their interactions with the human host. Application of genomic technologies in large, well-designed studies with detailed characterization of samples and metadata are required to disentangle these confounding effects. This Tanzanian case-control study, with complete metadata on a large number of participants, standardized phenotypic definitions and high-quality genotyping data, provides a solid platform for genomic discovery.

We sought to test, in a setting of high malaria transmission, the evidence for 40 relatively new loci (114 SNPs) that have been associated with severe malaria risk in 4 recent GWASs [3–6]. We confirmed the strong protective effects of the HbAS polymorphism, as well as the direct or indirect role of SNPs in 5 other loci (*USP38, FREM3, SDC1, DDC,* and *LOC727982*). We could not confirm that polymorphisms in the *ATP2B4* locus (rs10900585, rs4951074 [4], rs55868763, rs3753036, and rs1541255) were associated with severe malaria (*P* > .25). It is possible that the *ATP2B4* effect established in a West African population [4, 7] may be obscured by allelic heterogeneity in the genomic region which was not captured by the 5 SNPs genotyped.

The *DDC* locus was associated with severe malaria through a mechanism underlying the pathogenesis of cerebral malaria and, although the number of cases was low (n = 99) compared with other phenotypes, it demonstrates potentially strong genetic effects mediated through serotonin- and catecholamine-related pathways. The *DDC* SNP data, including rs10249420 with its heterozygous advantage effect, are consistent with those from analysis of cerebral malaria in a Gambian study [3]. Other studies may have missed this association by having too few cerebral malaria cases or lacking SNP coverage of the *DDC* locus [4, 5]. In the Gambian study, a significant association was found at rs1451375 (OR for AC vs other, 0.69; *P* = .00007), and we observe some evidence of association in our Tanzanian data (AC vs other for severe malaria, 0.674 [95% CI, .497–.914,;*P* = .011]; cerebral malaria, 0.484 [.269–.871; *P* = .011]).

A full genetic survey of *DDC* and its surrounding regions followed by association testing is required. Our data suggest that polymorphisms in *IZKF1* (60 kb from *DDC*) are unlikely to explain the associations with severe malaria, thereby reducing the amount of sequencing or genotyping required. *DDC* was found to be under balancing selection, a potential mechanism of maintaining protection from severe malaria in a population, while retaining minimal putative deleterious effects for the host. Balancing selection has also been observed at the *HBB* [8] and *G6PD* loci [11].

The rs4266246 polymorphism (*USP38,* chromosome 4) was associated with pleiotropic protection from acidosis, severe malarial anemia, and severe malaria per se. SNPs in *USP38* have been associated with susceptibility to asthma [20], but the gene lies in a 700-kb region close to malaria candidates in the *GYP-E/A/B* gene cluster as well as the *SMARCA5* [5] and *FREM3* loci. Several SNPs in *FREM3* were associated with acidosis and severe malaria in our study. A study comparing human and chimpanzee sequences identified *FREM3* as being under ancient balancing selection [8], but using the case-control data with no “ancient” ancestral comparison we found no evidence of such a mechanism operating on a shorter time scale. The rs11899121 (*SDC1)* and rs10188961 (*LOC727982)* polymorphisms were associated with respiratory distress and acidosis, respectively, as well as with severe malaria per se. Both loci are located in regions with sparse reporting of any GWAS signals. Further characterization of the genetic variation in these regions in Tanzanian individuals through sequencing could help impute other genotypes for more detailed SNP association analysis.

Our final statistical model included factors with direct or indirect genetic underpinning to assess the overall heritability of susceptibility to severe malaria and apportion this between the various protective loci. Our estimate that genetic differences accounted for approximately 22% of the variation in the severe malaria phenotype in the Tanzanian population is very is similar to an estimate from a more robust family-based study (25% [2]). Of particular interest, however, is the relative magnitude of the individual genetic contributions. The sickle HbAS polymorphism accounted for a nominal 6.3%, higher than reported elsewhere (2% [2]), and the combination of new loci—*USP38* (1.8%), *FREM3* (0.7%), *SDC1* (0.6%), *DDC* (0.8), and *LOC727982* (0.4%)—explain another further 4.3% of the variation. The contributions of the new loci tend to be greater than the established effects of ABO blood group (0.2%), α-thalassemia (0.3%), and G6PD (0.5%), making them enticing targets for further investigation, including functional studies.

Additional biological insights and genetic targets are likely to come from advances in sequencing technologies leading to whole-genome approaches, replacing GWAS chips that rely on LD to detect associations. The resulting whole-genome studies will not only assess genotype-phenotype associations but will also uncover protective effects by identifying the signatures of natural genetic selection left by malaria-driven human evolution. Further insights will come through applying sequencing technologies to host, parasite, and mosquito DNA and studying genetic interactions. As here, future studies will require large numbers of well characterized samples to disentangle the host genetic contribution to malaria susceptibility and provide robust biological insights that could lead to the development of much-needed disease control measures.

## Supplementary Data

Supplementary materials are available at *The Journal of Infectious Diseases* online (http://jid.oxfordjournals.org). Supplementary materials consist of data provided by the author that are published to benefit the reader. The posted materials are not copyedited. The contents of all supplementary data are the sole responsibility of the authors. Questions or messages regarding errors should be addressed to the author.

Supplementary Data
