# Supplementary material for: USP38, FREM3, SDC1, DDC, and LOC727982 Gene Polymorphisms and Differential Susceptibility to Severe Malaria in Tanzania
Source: J Infect Dis. 2015 Mar 24;212(7):1129–39. doi: 10.1093/infdis/jiv192 (PMC4559194; doi:10.1093/infdis/jiv192)
Supplement: Supplementary Data [file supp_212_7_1129__index.html]

USP38, FREM3, SDC1, DDC, and LOC727982 Gene Polymorphisms and Differential Susceptibility to Severe Malaria in Tanzania — USP38, FREM3, SDC1, DDC, and LOC727982 Gene Polymorphisms and Differential Susceptibility to Severe Malaria in Tanzania — Supplementary Data 

# *USP38, FREM3, SDC1, DDC,* and *LOC727982* Gene Polymorphisms and Differential Susceptibility to Severe Malaria in Tanzania

## Supplementary Data

Supplementary Data

**Files in this Data Supplement:**

- Supplementary Figure 1 - pdf file
- Supplementary Table 1 - docx file
