# Supplementary material for: USP38, FREM3, SDC1, DDC, and LOC727982 Gene Polymorphisms and Differential Susceptibility to Severe Malaria in Tanzania
Source: J Infect Dis. 2015 Mar 24;212(7):1129–39. doi: 10.1093/infdis/jiv192 (PMC4559194; doi:10.1093/infdis/jiv192)
Supplement: Supplementary Data [file supp_jiv192_jiv192supp_fig1.pdf]

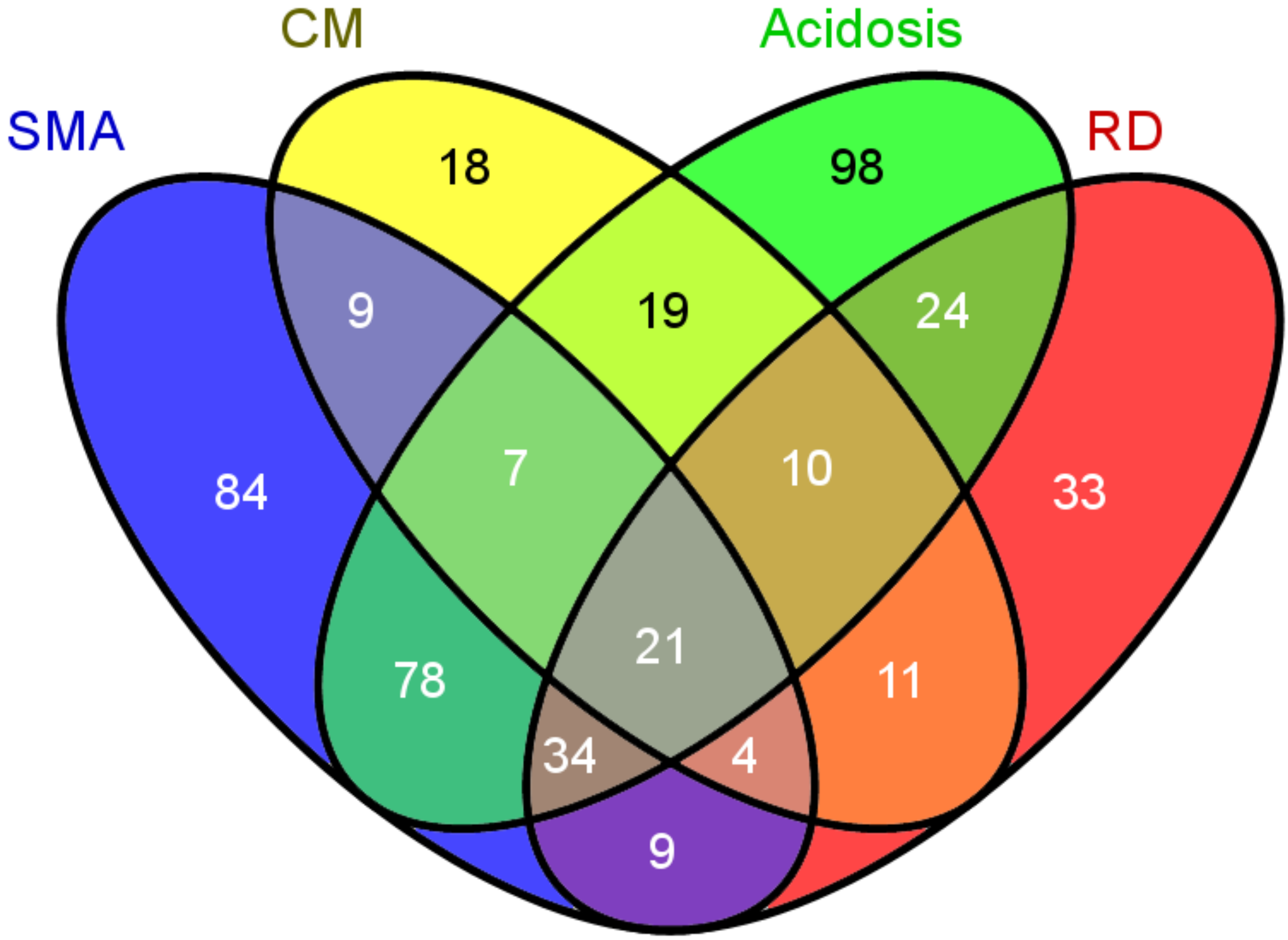

CM
Acidosis
SMA
RD
18
98
9
19
24
84
7
10
33
21
78
11
34
4
9

Supplementary Figure 1

The overlap of clinical phenotypes

SMA = severe malaria anaemia, CM = cerebral malaria, RD = respiratory distress
