# Supplementary material for: USP38, FREM3, SDC1, DDC, and LOC727982 Gene Polymorphisms and Differential Susceptibility to Severe Malaria in Tanzania
Source: J Infect Dis. 2015 Mar 24;212(7):1129–39. doi: 10.1093/infdis/jiv192 (PMC4559194; doi:10.1093/infdis/jiv192)
Supplement: Supplementary Data [file supp_jiv192_jiv192supp_table1.docx]

**Supplementary Table 1**

**SNPs genotyped**

| Chr | Position | Gene  name | RS  number | Ref.  allele | Source  * | Passed QC** |
| --- | --- | --- | --- | --- | --- | --- |
| 1 | 4817664 | *AJAP1* | kgp15825649 | A | [6] | Yes |
| 1 | 4834821 | *AJAP1* | rs6674631 | G | [6] | Yes |
| 1 | 81688714 | *LPHN2* | rs146428334 | A | [6] | No |
| 1 | 81726138 | *LPHN2* | rs72933304 | C | [6] | Yes |
| 1 | 81727427 | *LPHN2* | rs72933310 | T | [6] | Yes |
| 1 | 81751439 | *LPHN2* | rs72933350 | T | [6] | Yes |
| 1 | 81770827 | *LPHN2* | rs4650365 | C | [6] | Yes |
| 1 | 203652140 | *ATP2B4* | rs55868763 | C | [4] | Yes |
| 1 | 203652141 | *ATP2B4* | rs1541255 | A | [4] | Yes |
| 1 | 203654024 | *ATP2B4* | rs10900585 | G | [4] | Yes |
| 1 | 203660781 | *ATP2B4* | rs4951074 | G | [4] | Yes |
| 1 | 203677250 | *ATP2B4* | rs3753036 | G | [4] | Yes |
| 2 | 4901589 | *LOC727982* | rs1371478 | T | [6] | Yes |
| 2 | 4909777 | *LOC727982* | rs1371474 | C | [6] | Yes |
| 2 | 4926593 | *LOC727982* | rs10188961 | G | [6] | Yes |
| 2 | 20332487 | *LAPTM4A* | rs973128 | G | [6] | Yes |
| 2 | 20367973 | *SDC1* | rs11899121 | G | [6] | Yes |
| 2 | 188007364 | *ZSWIM2* | rs4316902 | A | [6] | Yes |
| 2 | 188012821 | *ZSWIM2* | rs144778284 | C | [6] | No |
| 3 | 16407519 | *OXNAD1* | kgp9483807 | C | [6] | No |
| 3 | 16408251 | *OXNAD1* | rs79691057 | G | [6] | No |
| 3 | 16408723 | *OXNAD1* | rs75180423 | C | [6] | No |
| 3 | 52231737 | *TLR9* | rs352140 | A | [4,6] | Yes |
| 3 | 160362359 | *ARL14* | rs76033371 | A | [6] | Yes |
| 3 | 160364808 | *ARL14* | rs75731597 | A | [6] | Yes |
| 3 | 160381509 | *ARL14* | rs74954675 | A | [6] | Yes |
| 3 | 160793678 | *B3GALNT1* | rs12107243 | C | [6] | Yes |
| 4 | 143538511 | *INPP4B* | rs77389579 | G | [5,6] | Yes |
| 4 | 143558581 | *INPP4B* | rs13103597 | C | [5,6] | Yes |
| 4 | 143971242 | *USP38* | rs4266246 | C | [5,6] | Yes |
| 4 | 144039139 | *USP38* | rs28459062 | T | [5,6] | Yes |
| 4 | 144261117 | *GAB1* | rs7663712 | A | [5,6] | Yes |
| 4 | 144540045 | *GUSBP5* | rs148111931 | T | [5,6,8] | No |
| 4 | 144665753 | *FREM3* | rs184908374 | G | [5,6,8] | No |
| 4 | 144666678 | *FREM3* | rs149914432 | A | [5,6,8] | Yes |
| 4 | 144680140 | *FREM3* | rs186790584 | A | [5,6,8] | Yes |
| 4 | 144698528 | *FREM3* | rs184895969 | C | [5,6,8] | No |
| 4 | 144702474 | *FREM3* | rs186873296 | A | [5,6,8] | Yes |
| 4 | 144948956 | *GYPB* | rs191338817 | A | [5,6,8] | No |
| 6 | 29588309 | *GABBR1* | rs192151845 | G | [6] | Yes |
| 6 | 29772098 | *HCG4* | rs114980857 | C | [6] | Yes |
| 6 | 31795550 | *HSPA1B* | rs6457452 | C | [6] | Yes |
| 6 | 31803074 | *SNORD48* | rs116288147 | C | [6] | Yes |
| 6 | 150942218 | *PLEKHG1* | rs55958968 | A | [3] | Yes |
| 6 | 150973623 | *PLEKHG1* | rs144224092 | C | [3] | No |
| 6 | 150975934 | *PLEKHG1* | rs79100774 | G | [3] | Yes |
| 6 | 150980481 | *PLEKHG1* | rs114726617 | G | [3] | Yes |
| 6 | 150981102 | *PLEKHG1* | rs2131263 | C | [3] | Yes |
| 6 | 150982529 | *PLEKHG1* | rs76924464 | G | [3] | Yes |
| 6 | 150994429 | *PLEKHG1* | rs151293197 | C | [3] | Yes |
| 6 | 151026346 | *PLEKHG1* | rs142712208 | G | [3] | Yes |
| 6 | 151046029 | *PLEKHG1* | rs15116938 | C | [3] | Yes |
| 6 | 151048708 | *PLEKHG1* | rs14155519 | C | [3] | Yes |
| 7 | 42445655 | *GLI3* | rs9942705 | G | [6] | Yes |
| 7 | 43283115 | *HECW* | rs17172181 | A | [6,9] | Yes |
| 7 | 43286129 | *HECW* | rs17172184 | G | [6,9] | Yes |
| 7 | 50418506 | *IZKF1* | rs7779749 | C | [3] | Yes |
| 7 | 50452552 | *IZKF1* | rs10230385 | A | [3] | Yes |
| 7 | 50460096 | *IZKF1* | rs6964823 | G | [3] | Yes |
| 7 | 50470059 | *IZKF1* | rs11552046 | G | [3] | No |
| 7 | 50470604 | *IZKF1* | rs4132601 | T | [3] | Yes |
| 7 | 50471613 | *IZKF1* | rs11980407 | A | [3] | Yes |
| 7 | 50473751 | *IZKF1* | rs6944602 | G | [3] | Yes |
| 7 | 50531681 | *DDC* | rs4947535 | A | [3] | Yes |
| 7 | 50532888 | *DDC* | rs11983581 | A | [3] | Yes |
| 7 | 50533062 | *DDC* | rs11982772 | A | [3] | Yes |
| 7 | 50534327 | *DDC* | rs11575527 | G | [3] | Yes |
| 7 | 50535395 | *DDC* | rs11575522 | C | [3] | Yes |
| 7 | 50535681 | *DDC* | rs11575518 | G | [3] | Yes |
| 7 | 50544663 | *DDC* | rs11575483 | C | [3] | Yes |
| 7 | 50567435 | *DDC* | rs11575387 | T | [3] | Yes |
| 7 | 50568735 | *DDC* | rs3779084 | A | [3] | Yes |
| 7 | 50570136 | *DDC* | rs880028 | G | [3] | Yes |
| 7 | 50572890 | *DDC* | rs6592961 | A | [3] | Yes |
| 7 | 50573333 | *DDC* | rs7809758 | G | [3] | Yes |
| 7 | 50574012 | *DDC* | rs1817074 | C | [3] | Yes |
| 7 | 50591583 | *DDC* | rs10271341 | T | [3] | Yes |
| 7 | 50605298 | *DDC* | rs11575320 | C | [3] | Yes |
| 7 | 50612202 | *DDC* | rs3779074 | G | [3] | Yes |
| 7 | 50612562 | *DDC* | rs2044859 | A | [3] | Yes |
| 7 | 50615440 | *DDC* | rs2329371 | G | [3] | Yes |
| 7 | 50620781 | *DDC* | rs6956737 | C | [3] | Yes |
| 7 | 50622712 | *DDC* | rs1451375 | C | [3] | Yes |
| 7 | 50623451 | *DDC* | rs10249420 | C | [3] | Yes |
| 7 | 50625898 | *DDC* | rs7803788 | C | [3] | Yes |
| 7 | 50629888 | *DDC* | rs6593010 | G | [3] | Yes |
| 7 | 50673171 | *DDC* | rs7800827 | C | [3] | Yes |
| 7 | 51824832 | *RP4* | rs662083 | T | [6] | No |
| 7 | 80302110 | *CD36* | CD36_G1439C | G | [22] | No |
| 7 | 80302115 | *CD36* | CD36_I1444D | I | [22] | No |
| 9 | 136131057 | *ABO* | rs150311214 | I | [2] | Yes |
| 9 | 136131064 | *ABO* | rs56390333 | G | [3,4] | No |
| 9 | 136131322 | *ABO* | rs8176746 | C | [3,4] | Yes |
| 9 | 136132909 | *ABO* | rs8176719 | I | [3,4] | Yes |
| 10 | 129975450 | *MKI67* | rs11016116 | A | [6] | Yes |
| 10 | 129976030 | *MKI67* | rs148494166 | T | [6] | No |
| 10 | 130072795 | *MKI67* | rs115947774 | A | [6] | No |
| 11 | 3847190 | *RHOG* | rs138826089 | T | [6] | No |
| 11 | 4111415 | *RRM1* | kgp12768002 | A | [6] | No |
| 11 | 5248173 | *HBB* | rs33950507 | G | [3,4] | No*** |
| 11 | 5248232 | *HBB* | rs334 | A | [3,4] | Yes |
| 11 | 5248233 | *HBB* | rs33930165 | G | [3,4] | No*** |
| 12 | 67366471 | *GRIP1* | rs192909543 | T | [6] | No |
| 12 | 67366537 | *GRIP1* | rs1394263 | T | [6] | Yes |
| 12 | 67369898 | *CAND1* | rs1566830 | G | [6] | Yes |
| 12 | 67394950 | *CAND1* | rs12307123 | C | [6] | Yes |
| 12 | 67455888 | *CAND1* | rs10459266 | C | [6] | Yes |
| 13 | 20050239 | *TPTE2* | rs182873742 | C | [6] | No |
| 14 | 75066093 | *LTBP2* | rs74063230 | G | [6] | No |
| 14 | 75274288 | *YLPM1* | rs10139016 | T | [6] | Yes |
| 14 | 75373034 | *RPS6KL1* | rs3742785 | A | [6] | Yes |
| 17 | 58855323 | *BCAS3* | rs184142841 | A | [6] | No |
| 17 | 59323072 | *TBX2* | rs73991577 | A | [6] | Yes |
| X | 153278307 | *IRAK1* | rs763737 | G | [6] | Yes |
| X | 153341060 | *MECP2* | rs1734792 | C | [6] | Yes |

* Reference number in main text, ** SNPs failed QC if they had more than 10% missing genotype calls or there was significant deviation from Hardy-Weinberg equilibrium (p<0.0001, autosomal only) or the minor allele frequency was less than 0.01; ** monomorphic as expected.
